# The Journal of the Gynæcological Society

**Published:** 1871-04

**Authors:** 


The Journal of the Gynæcological Society of Boston, a Monthly Journal devoted to the Advancement of the Knowledge of the Diseases of Women. Edited by Winslow Lewis, M.D., Horatio R. Storer, M.D., and George H. Bixby, M.D. Vol. III, July to January, 1870. For sale by Western News Co., Chicago. Price, $2.50.

We have fully commended the preceding volumes of this journal, and, in glancing over the present one, we find very much of great value to the practitioner. It is edited with much energy and spirit.

